# Modified SqueezeNet Architecture for Parkinson’s Disease Detection Based on Keypress Data

**DOI:** 10.3390/biomedicines10112746

**Published:** 2022-10-28

**Authors:** Lucas Salvador Bernardo, Robertas Damaševičius, Sai Ho Ling, Victor Hugo C. de Albuquerque, João Manuel R. S. Tavares

**Affiliations:** 1 Department of Software Engineering, Kaunas University of Technology, 51368 Kaunas, Lithuania; 2 Department of Electrical and Data Engineering, University of Technology Sydney, Sydney 2007, Australia; 3 Department of Teleinformatics Engineering, Federal University of Ceará, Fortaleza 60455-970, Brazil; 4 Instituto de Ciência e Inovação em Engenharia Mecânica e Engenharia Industrial, Departamento de Engenharia Mecânica, Faculdade de Engenharia, Universidade do Porto, 4200-465 Porto, Portugal

**Keywords:** Parkinson’s disease, neurodegeneration, early diagnosis, key typing, deep learning, convolutional network

## Abstract

Parkinson’s disease (PD) is the most common form of Parkinsonism, which is a group of neurological disorders with PD-like motor impairments. The disease affects over 6 million people worldwide and is characterized by motor and non-motor symptoms. The affected person has trouble in controlling movements, which may affect simple daily-life tasks, such as typing on a computer. We propose the application of a modified SqueezeNet convolutional neural network (CNN) for detecting PD based on the subject’s key-typing patterns. First, the data are pre-processed using data standardization and the Synthetic Minority Oversampling Technique (SMOTE), and then a Continuous Wavelet Transformation is applied to generate spectrograms used for training and testing a modified SqueezeNet model. The modified SqueezeNet model achieved an accuracy of 90%, representing a noticeable improvement in comparison to other approaches.

## 1. Introduction

Parkinson’s disease (PD) is a chronic and progressive movement disorder [1], being the second most prevalent neurodegenerative disorder in the world. It is believed that the disease affects over 1% of the world’s population, with its prevalence expected to double by 2030 [2]. The PD progression goes slowly, happening in several stages based on the affected brain region [3]. The disease’s early symptoms manifest in the disease’s middle stage, which correspond to a degeneration of 50%, or more, of the dopaminergic neuros in the Substantia Nigra part of the human brain [4].

In clinical environments, the common process of a PD diagnosis is performed based on the patient’s symptomatic history examination, where prodromal features, for instance, rapid eye movement, sleep disorder, hyposmia, and constipation; movement impairments, such as tremor, stiffness, and slowness [5]; psychological or cognitive disorders, such as, for example, anxiety, depression, and cognitive decline [6]; and family history, because 5 to 15% of the cases manifest in a family-related form [7], are taken into account.

With the progress of technology, each day more Artificial Intelligence (AI)-based solutions are allowing an automated recognition of many diseases [8,9,10], mainly a better understanding of PD [11], allowing the early diagnostics of the disease. Each year, numerous studies are published aiming to speed up the diagnosis [12] and management [13] of PD in order to allow a better patient quality of life. The motor symptoms characteristic of the disease affect simple daily activities performed by patients, such as typing on a computer’s keyboard [14,15]. In view of that, and aligned to the advances in AI-based solutions and current computational power, researchers [16,17,18] are aiming to find new approaches capable of detecting the motor impairments characteristic of PD [19].

In this study, we are focusing on the frequency aspect and how it influences the keyboard typing process by applying a convolutional neural network (CNN) on spectrograms generated from key-typing features of individuals belonging to two classes: subjects with PD and healthy (normal) individuals.

For the development of the proposed approach, the database [20] was applied as the data source. The presented database consists of 214 fields from which 202 features were extracted from 217 individuals from which 162 are PD patients and 55 are healthy individuals. Dekelly, et al. [20] divided the computer keyboard into three regions, *left* (L), *right* (R), and *space* (S), while measuring the pressure time on individual regions, such as the pressure on consecutive regions. From the collected values, 202 features, such as the flight time, mean pressure time, mean flight time, and others, were generated.

Due to the unbalanced nature of the dataset, a Synthetic Minority Oversampling Technique (SMOTE) was applied, increasing the volume of the data to 324. The values were then normalized using the *MinMax* technique and later applied to a Continuous Wavelet Transformer (CWT) in order to obtain the spectrogram of the data. The resulting images were applied to train a modified SqueezeNet model with seven fire modules and seven MaxPooling2D modules placed interleaved with each other.

The main contributions of this article are:

- A new approach for detecting Parkinson’s disease based on modifying a SqueezeNet CNN model.
- A new approach for detecting the symptoms of PD based on keyboard input, focusing on converting the acquired data into images by converting the input values into wavelets using a CWT in order to improve the results when detecting PD based on deep learning, which is an alternative approach to [21].

This article is organized into the following sections: Section 2, where similar works are described; Section 3, where the methods used are described; Section 4, where the obtained results and their assessment are presented; and Section 5, where the main conclusions are outlined and future work is suggested.

## 2. Related Works

In this section, works developed based on similar approaches aiming to detect PD are introduced and discussed.

Islam et al. [22] applied a four-convolutional and two fully connected layers model to obtain the detection of PD based on spiral drawing. The CNN layers were responsible for extracting the features of the used images and classifying them. The solution also applied data augmentation, which improved its performance. As a result, they obtained an accuracy of 96.64%.

On PD patients, the neurons of the SN region are deprived of their neuronal functions, which causes a striatal dopamine deficiency. Thus, Sivaranjini et al. [23] proposed the usage of Magnetic Resonance Imaging (MRI), which is able to capture the structural changes in the brain caused by a dopamine deficiency, to train an AlexNet CNN model. The proposed solution achieved 88.9% accuracy.

Gait information was used in [24] with a 1D CNN model (1D-Convnet) in order to build a deep neural network (DNN) classifier. The proposed classifier processed 18 1D signals originating from foot sensors attached to the subjects, which were responsible for measuring the vertical ground reaction force (VGRF). The first part of the solution consisted of 18 parallel 1D-Convnets that represented the system’s input. The second part was a fully connected network responsible for merging the 18 signals in order to obtain a final classification. As a result, 98.7% accuracy was obtained.

Shivangi et al. [25] applied VGFR spectrum detector and voice impairment classifier models to detect PD in its early stages. As input, two different approaches were tested. The first one used as an input was a series of spectrograms generated from gait signals, and in the second approach, a deep dense artificial neural network (ANN) of a voice recording was used. The experiments performed demonstrated a higher accuracy for the ANN-based approach, achieving an accuracy of 89.1% for the voice impairment classifier, while the gait spectrogram images resulted in 88.1% accuracy.

A hybrid two-stage approach for detecting PD was proposed in [26]. The proposed solution counted 514 spiral drawings made by PD-affected and healthy individuals. For the purpose of diagnosis, a SqueezeNet CNN model was applied for extracting features of the drawings, which were later classified using a Support Vector Machine (SVM). As a result, the solution obtained 91.26% accuracy, leading to a good improvement when compared to other solutions explored in the study.

In [27], CNN data acquired using sensors from the spiral drawing movement process were applied. The inputs for the proposed CNN model consisted of a module of an applied Fast Fourier’s transform (FFT) in the range of frequencies between 0 and 25 Hz. The discrimination capability of different directions during the drawing movements on the X and Y axes were analyzed to establish the best result. For its best result, the solution presented 96.5% accuracy.

The HandPd dataset, which contains images of handwritten spirals and a Meander template of PD and healthy individuals, was used by [28] in order to detect Parkinson’s disease in its early stages. The images were applied to a transfer learning–deep learning process which aimed to detect the disease. The proposed method obtained 98.24% accuracy on the spiral image set and 98.11% on the Meander set.

In [29], three machine learning algorithms were applied on the collected data from the spiral drawing process using a computer mouse in order to detect PD in its early stages. To achieve this goal, three selected drawings were chosen, the Archimedes spiral, triangle, and cube. As a result, 96% accuracy was obtained for the triangle drawing, 100% for the cube drawing, and 100% for the spiral drawing and 100% sensitivity on all three patterns. However, due to the small size of the dataset, the perfect results might have been a result of over-fitting.

Handwritten spiral and wave drawings made by PD and healthy individuals were used in [30] to train and test CNN models. The used dataset was composed of drawings made by 55 individuals. As a result, 93.3% accuracy was obtained.

To detect PD, in [31], a Leap Motion device was used to acquire motion data from volunteers while performing three motor tasks: finger tapping, finger opening–closing, and pronation–supination of the hands. The input data were then used to train a one-dimensional (1D) CNN model. The features learned by the CNN model were then applied to three machine learning algorithms, mainly the KNN, SVM, DT, and RF. For its best result, the solution achieved 85.1% accuracy.

In [32], a VGG-19 model was applied to detect PD. To train and test the proposed solution, the Kaggle dataset was used, which contains 102 images of handwritten spirals and 102 images of handwritten waves. The data were submitted to the process of pre-processing, where they were resized and passed through a process of data augmentation. As a result, the proposed solution achieved 88% accuracy and 89% sensitivity.

A new technique to detect PD based on wavelets-extracted features and machine learning paradigms was presented in [16]. To achieve the aimed goal, the volunteers, Parkinson’s patients and healthy individuals, were requested to perform typing tasks, from which the flight time and hold time of each pressed key were used to generate the used dataset. As a result, the study reported 100% accuracy.

Discriminating visual clues were extracted using CNN models from handwritten data in [33] in order to detect PD. The proposed solution obtained 83% accuracy, proving to be a good solution for the problem under study.

In [34], an accuracy of more than 87% was obtained when detecting PD based on handwriting dynamic data acquired from individuals when submitted to tasks defined to measure their abilities related to writing skills by applying deep learning architectures.

The work presented in [35] used voice signals for detecting PD by applying 18 features extraction techniques on the input signals, which were obtained from two microphone channels of an acoustic cardioid and a smartphone. The proposed solution obtained 94.55% accuracy in its best performance.

In summarizing, even with the high number of studies developed every year and countless efforts to detect PD automatically, many models fail when applied in a real-life environment, due to the complexity and variety of the disease symptoms. Table 1 offers an overview of the results obtained by the identified similar studies.

## 3. Methodology

In this section, the theoretical and methodological concepts needed to better understand the proposed solution are presented.

### 3.1. Workflow

**Figure 1:**
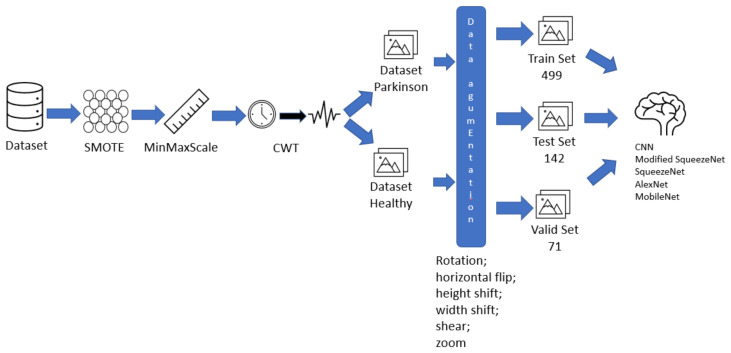
Fluxogram of the proposed solution.

The main steps for the development of this study are presented in Figure 1, which offers a visual summary of the procedures taken in this study.

The steps are summarized below:

1. Data preparation, with features selection, data balancing with SMOTE technique, and data isolation (Section 3.2 and Section 3.3).
2. Image generation applying the Continuous Wavelet Transform (Section 3.4).
3. The modified SqueezeNet structure is presented (Section 3.7).

### 3.2. Dataset

**Figure 2:**
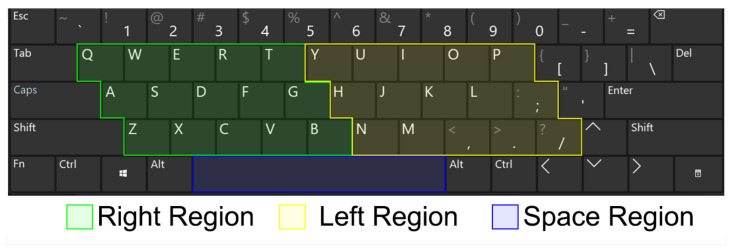
Keyboard collection structure used to build the used experimental dataset.

*Keyboard Taps Data from Kaggle and MIT* is a dataset developed by [21], composed of key-pressing data collected from 227 healthy and PD-affected individuals from Canada, United States, Australia, and United Kingdom. The author divided the computer keyboard into 3 regions: the left region (L), right region (R), and space bar region (S), Figure 2.

**Figure 3:**
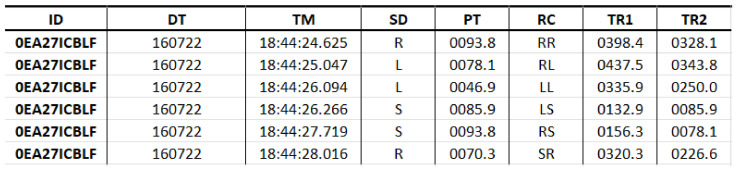
Structure of the used dataset.

The software developed in [21], *Tappy*, kept tracking the normal usage of the computer’s keyboard by the volunteers of the study, storing the input data in text files, Figure 3, which stored the individual’s identification code (ID), the date (DT) and time (TM) when the key was pressed, the pressing region (SD), the hold time (PT) in milliseconds (ms), the region change (RC), the latency time (TR1), the flight time between subsequent keys (TR2). Other features which were not considered for this study included disease manifestation side, body side which is most affected by the disease motor symptoms, and the disease stage according to the Unified Parkinson’s Disease Rating Scale (UPDRS).

For this study, an updated version based on the base structure of the database was used, where 202 features were generated from regions L, R, and change in regions based on the descriptive statistics, such as mean, standard deviation, and kurtosis, to generate the following features:

- Flight time mean;
- Flight time standard deviation;
- Flight time kurtosis;
- Flight time skew;
- Flight time percentile 10th;
- Flight time percentile 20th;
- Flight time percentile 40th;
- Flight time percentile 60th;
- Flight time percentile 70th;
- Flight time percentile 80th;
- Flight time percentile 90;
- Hold time mean;
- Hold time standard deviation;
- Hold time kurtosis;
- Hold time skew;
- Hold time percentile 10th;
- Hold time percentile 20th;
- Hold time percentile 40th;
- Hold time percentile 60th;
- Hold time percentile 70th;
- Hold time percentile 80th;
- Hold time percentile 90th;
- Latency time mean;
- Latency time standard deviation;
- Latency time kurtosis;
- Latency time skew;
- Latency time percentile 10th;
- Latency time percentile 20th;
- Latency time percentile 40th;
- Latency time percentile 60th;
- Latency time percentile 70th;
- Latency time percentile 80th;
- Latency time percentile 90th;
- Total count;
- Mean difference L/R hold time;
- Mean difference LR/RL latency time;
- Mean difference LL/RR latency time.

Those values were then stored as a *csv* file for the image generation process.

### 3.3. SMOTE

Unbalanced data are a recurrent issue in classification tasks, being found in numerous applications [37]. To balance the data and overcome eventual issues caused by such nature of the input set techniques, such as the Synthetic Minority Oversampling Technique (SMOTE), are applied.

SMOTE generates data for the minority class by applying a K-nearest neighbors (K-NN) approach, although, differently from other KNN-based oversampling approaches where the synthetic values are randomly generated directing to its k-nearest neighbors, the SMOTE technique consists of assigning weights to each neighbor direction, assigning smaller weights for positions that can generate over generalization [38].

Mathematically, the SMOTE technique can be expressed by a given minority class *A*, where for each x∈A the Euclidean distance between *x* and the other samples of *A* are extracted. Later, a set for samples from *N* are randomly chosen from its K-nearest neighbors, constructing a set $A_{1}$.

For each $x_{k}\;inA_{1}$, Equation (1) is applied to generate a new oversampled set.

(1) $$x^{\prime }=x+rand\left(0,1\right)*\left|x-x_{k}\right|$$

### 3.4. Continuous Wavelet Transform

Continuous Wavelet Transform (CWT) offers a straightforward approach for visualizing the signal behavior on frequency domain; CWT consists of a correlation measure between a signal and multiple wavelets deriving from the base one. It can be obtained by changing the size of the analysis window, translating it on time, multiplying it by the input signal, and integrating it across the time intervals. We can express such concept mathematically by Equation (2), where $d_{Z}\left(a,b\right)$ represents the wavelet coefficient of the continuous variable Z={Z(t),t∈R} for a given scale *a* and shift *b*.

(2) $$d_{Z}\left(a,b\right)=\frac{1}{\sqrt{a}}\int_{R}\psi \left(\frac{t}{a}-b\right)Z\left(t\right)dt=<\psi_{\lambda },Z{>}_{L^{2}\left(R\right)}$$

For discrete input signals, the discretized wavelet coefficient $e_{Z}\left(a,b\right)$ can be obtained by applying the Riemman sum. Equation (3) is obtained, in such cases, when the function ψ satisfies $M\in \int e^{*}$.

(3) $$\begin{array}{c} \int_{R}t^{m}\psi \left(t\right)dt=0\;\;\mathrm{for}\;\mathrm{all}\;m\in \{0,1,\ldots ,M\} \end{array}$$

Given a signal x(t), its CWT is given by Equation (4), where $W\left(\lambda ,t\right)$ is the wavelet coefficient. ψ(t) corresponds to the functional form of the reference wavelet and $\psi^{*}$ its complex conjugate, λ corresponds to the scale responsible for changing the frequency being measured by a given wavelet.

(4) $$W\left(\lambda ,t\right)=\int_{-\infty }^{\infty }x\left(\tau \right)\psi_{\lambda ,t}^{*}\left(\tau \right)d\tau .$$

The functional form of the mother wavelet ψ can be represented by Equation (5)

(5) $$\psi_{\lambda ,t}\left(\tau \right)=\frac{1}{\sqrt{\lambda }}\psi \left(\frac{\tau -t}{\lambda }\right)$$

### 3.5. Convolutional Neural Network

**Figure 4:**
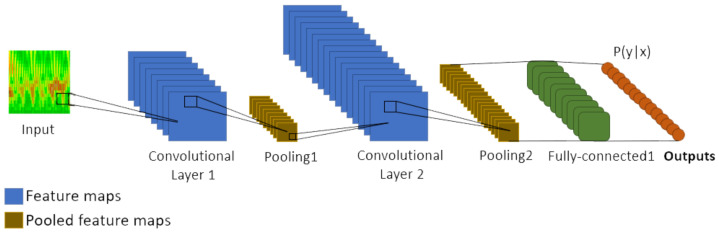
Common architecture of a Convolutional Neural Network.

In a standard manner, a CNN, Figure 4, has an input layer, three convolutional layers, two max-pooling layers, and one fully connected layer. An input layer receives the image from which the pixels contribute to the output through a set of kernel filters, also known as weights or mask. Typically, the applied filter is fixed, depending on the specified network layer, the output being a result of bi-dimensional convolutional operations:

(6) U=conv2d(X,W)

where *X* corresponds to the input image, *U* the output image, and *W* the weight matrix or 2D filter matrix.

A non-linear function σ is also added to the linear part of *U* layer, in that manner the output is obtained:

(7) $$z_{mn}=\sigma (u_{mn}+b),\;\mathrm{or}\;\;Z=\sigma \left(W⋆X+B\right)$$

where σ represents the *d* activation function such as Sigmoid, *B* represents the bias for the layer, and *Z* represents a feature map.

For a given 2D image (*I*), the convolution of the feature map (*X*) by the weight matrix (*W*) in a given point (I(p,q)) is:

(8) $$U_{p,q}={\left(W⋆X\right)}_{p,q}=\sum_{m=0}^{r-1}\sum_{n=0}^{c-1}W_{m,n}X_{p-m,q-n}$$

### 3.6. SqueezeNet

Mentioned for the first time in [39], SqueezeNet consists of a network designed with an architecture 50 times smaller than AlexNet network, although equally powerful and 3 times faster. SqueezeNet is vastly used in medical field due to its performance and fast execution, as we can see in [40].

**Figure 5:**
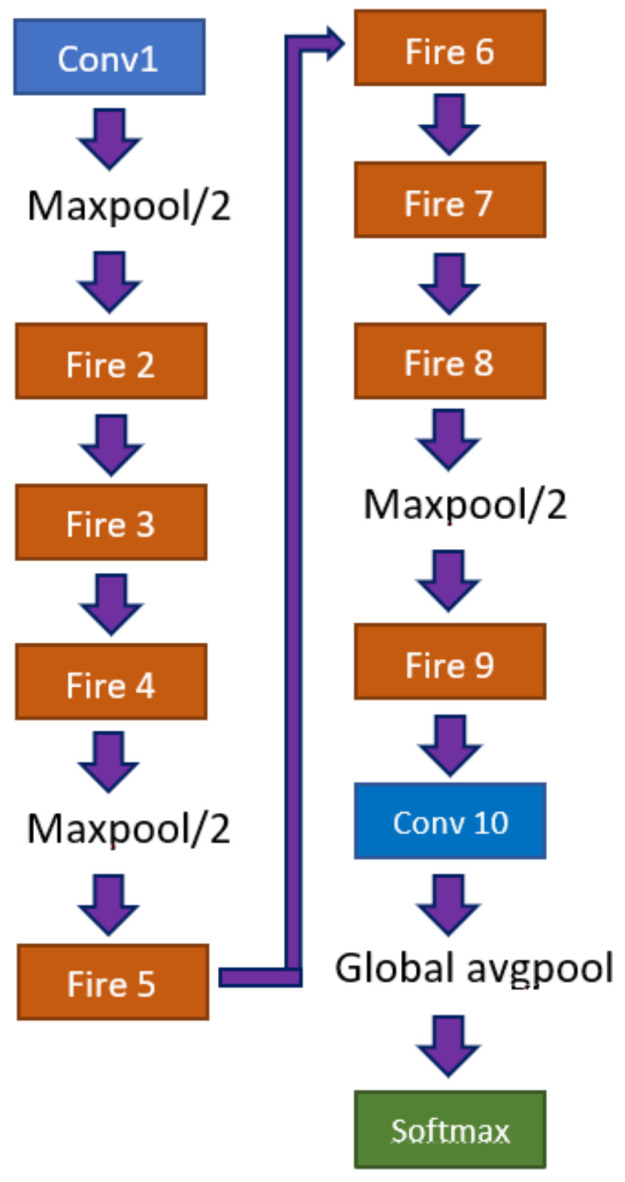
Architecture of a SqueezeNet model.

A SqueezeNet model is composed of a standalone convolutional layer, responsible for receiving the input image, followed by 8 fire modules, and ending with a convolutional layer, Figure 5. The network is characterized by a series of “squeeze”, composed by 1×1 filters, and “expand”, composed by a series of 1×1 and 3×3 filters, layers. The joint of both layers is named Fire module.

### 3.7. Modified SqueezeNet

**Figure 6:**
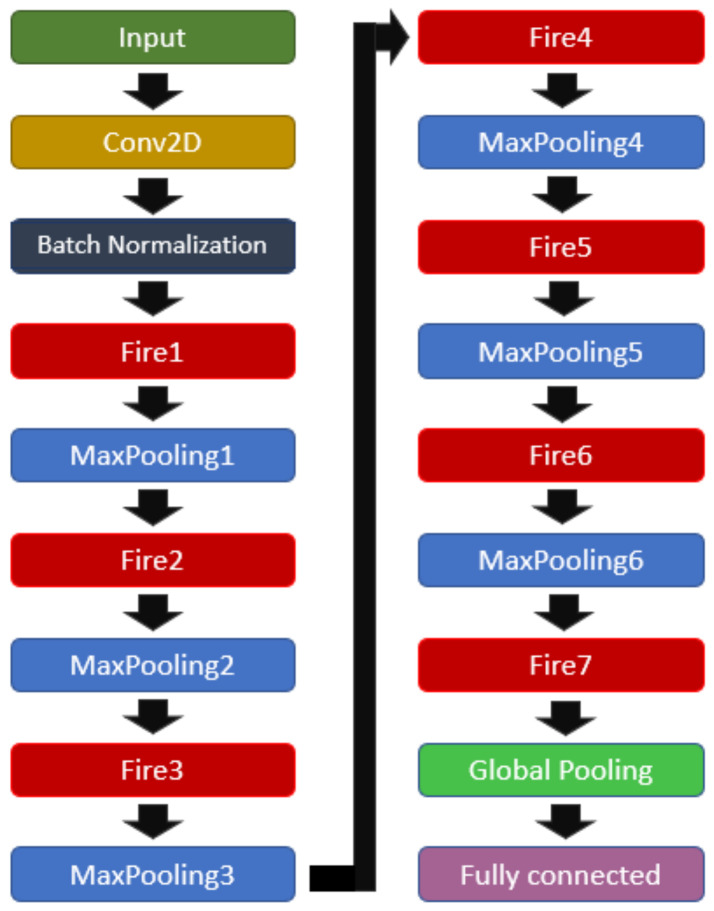
Modified SqueezeNet structure.

During tests applying a SqueezeNet model, it was observed that such architecture outperformed other applied models; however, the resulted metrics still presented inferior values than those desired. In view of that, a set of trials was performed in changing the original SqueezeNet architecture, aiming to maximize the performance of the network. As a result of such trials, an improved architecture composed of 1 input layer, 1 batch normalization layer, 7 Fire modules, Table 2, 6 pooling layers with a pool size of 2×2, 1 global pooling layer, and 1 fully connected layer was generated(see Figure 6).

### 3.8. Performance Evaluation

To evaluate the performance of the proposed solution, we use the confusion matrix for the binary classification problem under study, which is presented in Table 3.

Many performance measures can be obtained from the confusion matrix, such as:

Sensitivity, which is the rate of data correctly classified as positive observations;

Specificity, which is the rate of data correctly classified as negative observations.

F1-Score is applied to establish the performance of a binary classifier as the harmonic mean of precision (PPV) and recall.

The validation loss (valid loss) is obtained by running the neural network forward over the inputs ($x_{i}$), comparing the outputs ($\hat{y_{i}}$) with the true values, i.e., the ground-truth values, ($y_{i}$) by applying a loss function defined as:

(9) $$J=\frac{1}{N}\sum_{N}^{i=1}L\left(\hat{y_{i}},y_{i}\right)$$

where *L* represents the individual loss function based on the differences in predicted and target values, and *N* the number of generated outputs.

## 4. Experiments and Results

This section will approach the computational and mathematical experiments performed for the development of this work.

### 4.1. Data Preparation

The dataset was stored in a csv file, with 215 columns containing information, such as the subject’s identification, and attributes, such as gender, state of Parkinson’s, tremors, and diagnosis year. Initially, those values are excluded from the set of features from the collection, once only the numerical values are relevant for this study.

**Figure 7:**
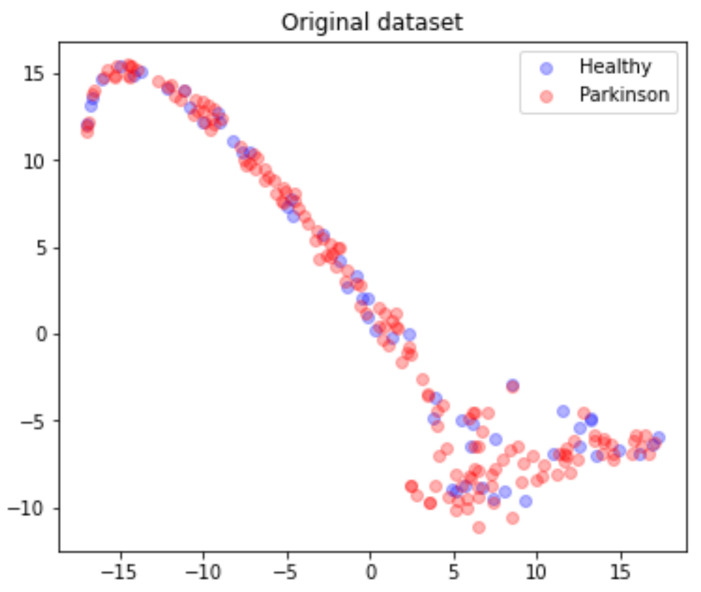
Dataset points distribution according to health status.

The 201 data rows, each belonging to different individuals, are mapped and converted into a single matrix M(217,202), and the matrix’s points distribution can be seen in Figure 7.

**Figure 8:**
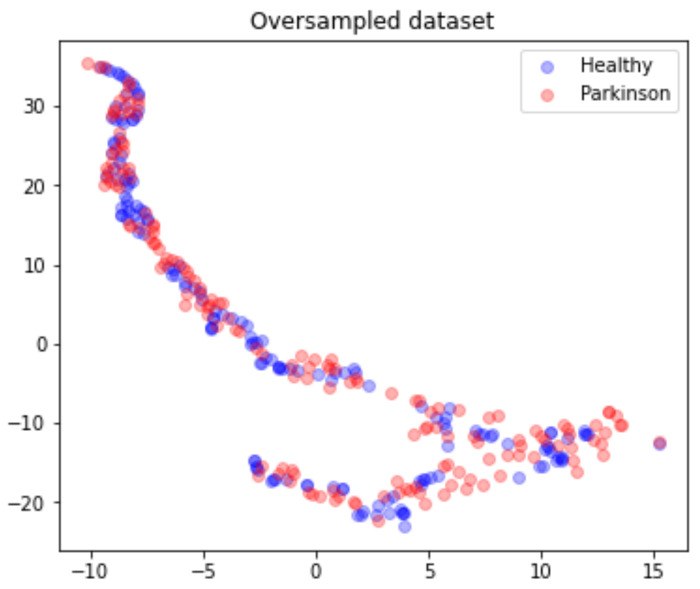
Oversampled dataset points distribution according to health status.

Due to the highly unbalanced nature of the data, the matrix *M* is submitted to an oversampling process where a SMOTE technique was applied with a random generation seed of 42. This process results in a matrix M(321,212), from which 160 are Parkinson’s and 161 are healthy, as presented in Figure 8.

### 4.2. Image Dataset

**Figure 9:**
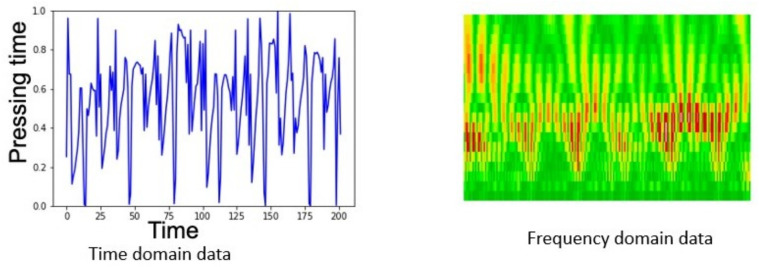
Examples of time and frequency domain data in the dataset.

The rows of matrix *M* are then isolated and separated into two sets of vectors, healthy and parkinson, which are normalized with a *MinMaxScale* ranging the values in the interval [0,1]. The vectors are then applied to the image generation by applying scales in the interval A=[0,20] and the Morlet Wavelet following Equation (10). The sample images of the time domain and frequency domain data can be seen in Figure 9.

(10) $$\psi \left(t\right)=exp^{-\frac{t^{2}}{2}}cos\left(5t\right)$$

To increase the volume of the data, and thus improving the precision of the model, the newly generated images are submitted to a data augmentation process, where the morphological transformations *rescale*, *rotation* at the 0 to 40 range, *horizontal flip*, 0 to 0.2 *height shift* range, *shear* at 0 to 0.2 range, and *zoom* ranging from 0 to 0.2. Those processes increased the input data volume from 217 to 712 images in both classes. The data were then split into three datasets, *train*, *test*, and *valid*, used for training, testing, and validating the model.

### 4.3. Experimental Setting

For the development of this work, a Colab GPU, with 13 GB of RAM, running Ubuntu 18.04.3 LTS, was used. For the implementation of the proposed solution, the Python 3 programming language was used, and to assess its efficiency, metrics such as accuracy, recall, F1-Score, and the binary confusion matrix were adopted.

### 4.4. Training

**Figure 10:**
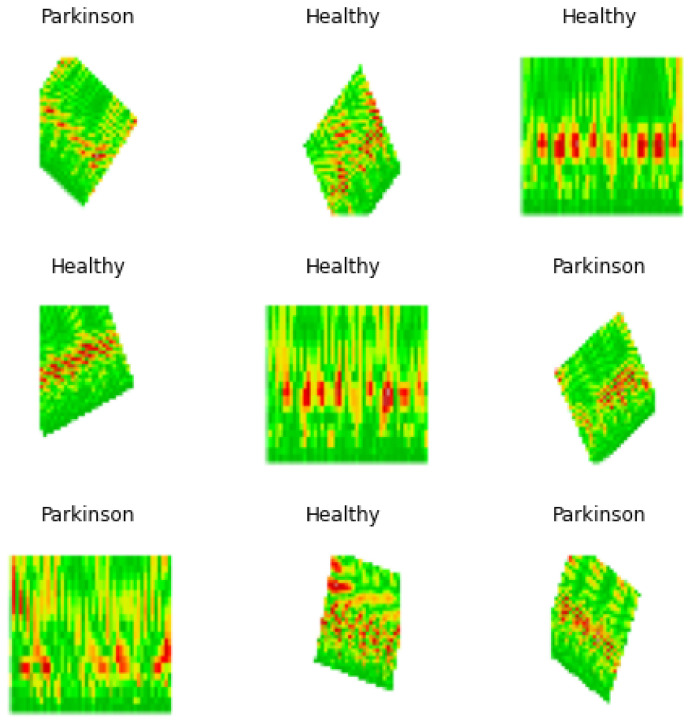
Batch sample of images used for model training.

**Figure 11:**
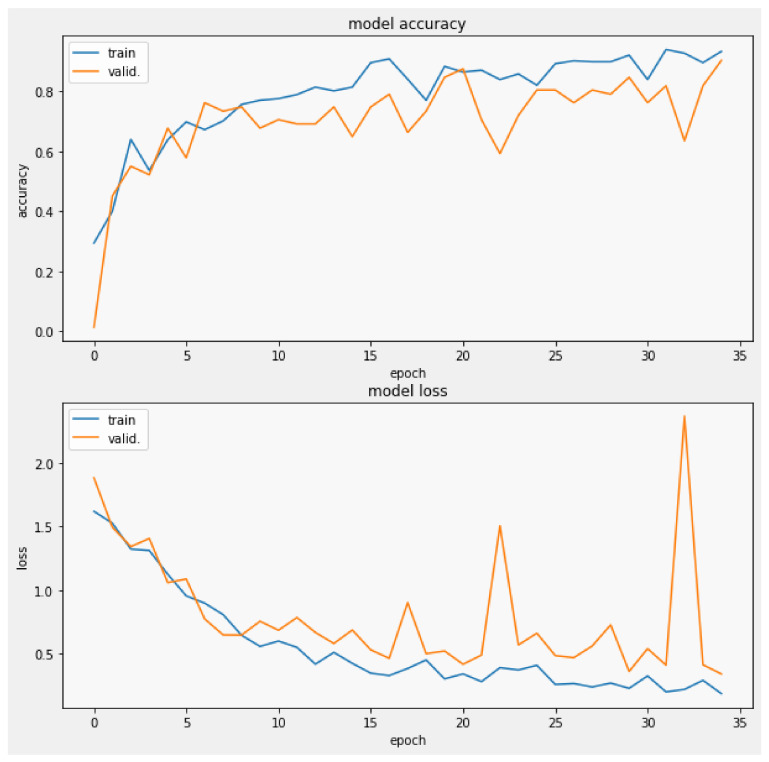
Validation loss and model accuracy by epoch during the training process.

The training process was performed applying a batch size of 8 with images of 240×240×3, Figure 10, performing 20 steps per epoch in 35 epochs and applying the *early stop by valid loss* method. This measures the validation loss obtained during each epoch, stopping the process of training once the valid loss value reached a point lower than the established threshold of 0.4, where the model reached an accuracy of 90% with a valid loss of 0.3353, which was plotted in Figure 11, from which was possible to observe the convergence of the valid loss and train loss during the training process.

### 4.5. Results

**Figure 12:**
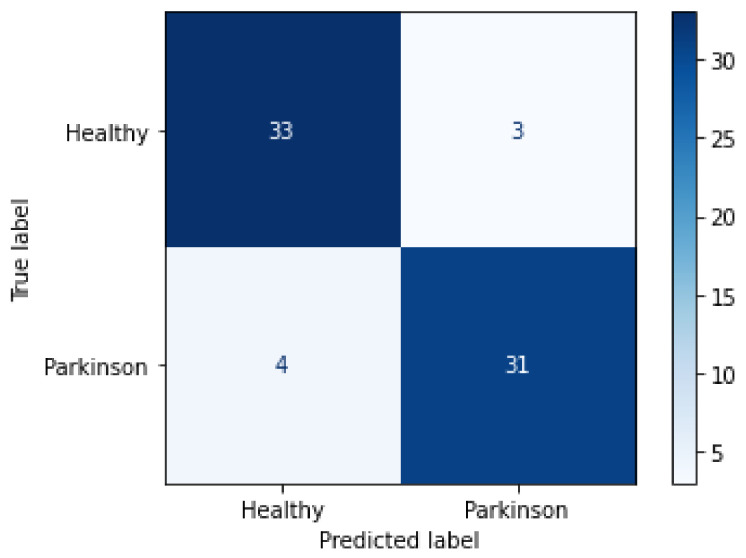
Confusion matrix for the validation process.

For an easier view of the individual performance by class, the obtained metrics were placed in Table 4 and later applied for the construction of the confusion matrix, Figure 12.

From the confusion matrix, it was possible to observe that from the 71 images applied to test the final trained model, 35 belonging to the healthy class were correctly classified, while 9 were wrongly classified as Parkinson’s disease. On the other hand, only 5 of the 35 images belonging to the Parkinson’s disease class were wrongly classified as healthy, while 30 were correctly detected, thus achieving, on average, 90% accuracy with a 95% confidence interval of [0.22, 0.4] and a mean of 0.3.

### 4.6. Comparison of Results

To compare the efficiency of our proposed model, which achieved 90% accuracy when submitted to the validation set (validation accuracy), three other CNN models were trained and validated, being SqueezeNet which achieved an accuracy of 72.53%, AlexNet with an accuracy of 76.76%, and MobileNet V3 with an accuracy of 76.56%. The results are summarized in Table 5.

To assess the influence of the SMOTE technique in the proposed solution’s result, the values with and without the SMOTE technique were compared, and the results are presented in Table 6. The results show that SMOTE has allowed to improve the performance of the classification.

## 5. Conclusions

This study showed that altering the original structure of the SqueezeNet architecture, aligned with the usage of the Continuous Wavelet Transform (CWT) for image generation, can significantly improve the process of detecting Parkinson’s disease. The results of the current study displayed that in terms of the used accuracy metrics, the intrinsic features can be observed within the input key-typing data [21] which are responsible for the good achieved performance. Therefore, a simple task such as key typing can help in the diagnosis of Parkinson’s disease which affects millions of people worldwide.

## Figures and Tables

**Table 1:** Summary of the identified related work.

Reference	Input Data	Algorithms	Accuracy
[22]	Spiral drawing	CNN	96.64%
[23]	MRI images	AlexNet	88.9%
[24]	Sensors signals	DNN	98.7%
[25]	Voice impairment	ANNs	89.1%
[26]	Spiral drawing	SqueezeNet	91.26%
[27]	Sensors signals	CNN	96.5%
[28]	Spiral drawing	CNN	98.24%
[29]	Spiral drawing	ML	96%
[30]	Drawing	CNN	93.3%
[31]	Leap Motion	CNN	85.1%
[32]	Spiral drawing	VGG-19	88%
[16]	Typing tasks	ML	100%
[33]	Handwritten	CNN	83%
[36]	Hand-drawn	FOPF	74.63%
[34]	Handwritten	Deep Learning	87%

**Table 2:** Fire modules.

Fire Module	Squeeze Size	Expand Size
Fire 1	24	48
Fire 2	48	96
Fire 3	64	128
Fire 4	64	256
Fire 5	64	128
Fire 6	48	96
Fire 7	24	48

**Table 3:** Confusion matrix built for the binary classification problem under analysis.

	True Class	+1	−1
Prediction			
+1		TP	FP
−1		FN	TN

Here, *TP* represents the number of true-positive samples, *TN* represents the number of true-negative samples, *FP* represents the number of false-positive samples, and *FN* represents the number of false-negative samples.

**Table 4:** Summary of classes performance.

Class	Accuracy	Precision	Recall	F1-Score
Healthy	89%	89%	92%	90%
Parkinson	91%	89%	89%	90%

**Table 5:** Comparison of obtained performance metrics.

Model	Accuracy	Precision	Recall	F1-Score	Training Epochs
AlexNet	76%	77%	68%	72%	49
SqueezeNet	72%	68%	71%	70%	26
MobileNet V3	60%	54%	76%	63%	49
Modified SqueezeNet (proposed)	90%	89%	91%	90%	35

**Table 6:** Comparison SMOTE technique.

SMOTE Status	Epochs	Accuracy	Precision	Recall	F1-Score
With SMOTE	35	90%	89%	91%	90%
Without SMOTE	49	76%	77%	68%	72%

## Data Availability

Data used in this investigation are available under the ODC Public Domain Dedication and License from the PhysioNet repository at https://physionet.org/physiobank/database/tappy/ (accessed on 16 June 2022).

## References

[1] Abdulhay E., Arunkumar N., Narasimhan K., Vellaiappan E., Venkatraman V. (2018). Gait and tremor investigation using machine learning techniques for the diagnosis of Parkinson disease. Future Gener. Comput. Syst..

[2] Aarsland D., Batzu L., Halliday G.M., Geurtsen G.J., Ballard C., Chaudhuri K.R., Weintraub D. (2021). Parkinson disease-associated cognitive impairment. Nat. Rev. Dis. Prim..

[3] Braak H., Tredici K.D., Rüb U., de Vos R.A., Steur E.N.J., Braak E. (2003). Staging of brain pathology related to sporadic Parkinson’s disease. Neurobiol. Aging.

[4] Sy M.A.C., Fernandez H.H. (2020). Pharmacological Treatment of Early Motor Manifestations of Parkinson Disease (PD). Neurotherapeutics.

[5] Nemade D., Subramanian T., Shivkumar V. (2021). An Update on Medical and Surgical Treatments of Parkinson’s Disease. Aging Dis..

[6] Armstrong M.J., Okun M.S. (2020). Diagnosis and Treatment of Parkinson Disease. JAMA.

[7] Balestrino R., Schapira A. (2019). Parkinson disease. Eur. J. Neurol..

[8] Park C., Ha J., Park S. (2020). Prediction of Alzheimer’s disease based on deep neural network by integrating gene expression and DNA methylation dataset. Expert Syst. Appl..

[9] Desai M., Shah M. (2021). An anatomization on breast cancer detection and diagnosis employing multi-layer perceptron neural network (MLP) and Convolutional neural network (CNN). Clin. eHealth.

[10] Sekeroglu B., Ozsahin I. (2020). Detection of COVID-19 from Chest X-ray Images Using Convolutional Neural Networks. SLAS Technol..

[11] Espay A.J., Bonato P., Nahab F.B., Maetzler W., Dean J.M., Klucken J., Eskofier B.M., Merola A., Horak F., Lang A.E. (2016). Technology in Parkinson’s disease: Challenges and opportunities. Mov. Disord..

[12] Loh H.W., Hong W., Ooi C.P., Chakraborty S., Barua P.D., Deo R.C., Soar J., Palmer E.E., Acharya U.R. (2021). Application of deep learning models for automated identification of parkinson’s disease: A review (2011–2021). Sensors.

[13] Mughal H., Javed A.R., Rizwan M., Almadhor A.S., Kryvinska N. (2022). Parkinson’s Disease Management via Wearable Sensors: A Systematic Review. IEEE Access.

[14] Ulinskas M., Woźniak M., Damaševičius R. (2017). Analysis of Keystroke Dynamics for Fatigue Recognition. Computational Science and Its Applications—ICCSA 2017.

[15] Ulinskas M., Damaševičius R., Maskeliunas R., Woźniak M. (2018). Recognition of human daytime fatigue using keystroke data. Procedia Comput. Sci..

[16] Peachap A.B., Tchiotsop D., Louis-Dorr V., Wolf D. (2020). Detection of early Parkinson’s disease with wavelet features using finger typing movements on a keyboard. SN Appl. Sci..

[17] Barnardo L.S., Damasevicius R., Maskeliunas R. (2022). Using Keytyping as a Biomarker for Cognitive Decline Diagnostics: The Convolutional Neural Network Based Approach. Mediterranean Conference on Pattern Recognition and Artificial Intelligence.

[18] Tripathi S., Arroyo-Gallego T., Giancardo L. (2022). Keystroke-Dynamics for Parkinson’s Disease Signs Detection in An At-Home Uncontrolled Population: A New Benchmark and Method. IEEE Trans. Biomed. Eng..

[19] Alfalahi H., Khandoker A.H., Chowdhury N., Iakovakis D., Dias S.B., Chaudhuri K.R., Hadjileontiadis L.J. (2022). Diagnostic accuracy of keystroke dynamics as digital biomarkers for fine motor decline in neuropsychiatric disorders: A systematic review and meta-analysis. Sci. Rep..

[20] Dekelly P. (2018). Tappy Keystroke Data with Parkinson’s Patients. https://www.kaggle.com/code/yoavben/predicting-parkinson-s-disease-from-keyboard-data/data.

[21] Adams W.R. (2017). High-accuracy detection of early Parkinson’s Disease using multiple characteristics of finger movement while typing. PLoS ONE.

[22] Islam M.R., Matin A., Nahiduzzaman M., Siddiquee M.S., Hasnain F.M.S., Shovan S.M., Hasan T. (2021). A Novel Deep Convolutional Neural Network Model for Detection of Parkinson Disease by Analysing the Spiral Drawing. Proceedings of the International Joint Conference on Advances in Computational Intelligence.

[23] Sivaranjini S., Sujatha C.M. (2019). Deep learning based diagnosis of Parkinson’s disease using convolutional neural network. Multimed. Tools Appl..

[24] Maachi I.E., Bilodeau G.A., Bouachir W. (2020). Deep 1D-Convnet for accurate Parkinson disease detection and severity prediction from gait. Expert Syst. Appl..

[25] Shivangi, Johri A., Tripathi A. Parkinson Disease Detection Using Deep Neural Networks. Proceedings of the 2019 Twelfth International Conference on Contemporary Computing (IC3).

[26] Bernardo L.S., Damaševičius R., de Albuquerque V.H.C., Maskeliūnas R. (2021). A hybrid two-stage SqueezeNet and support vector machine system for Parkinson’s disease detection based on handwritten spiral patterns. Int. J. Appl. Math. Comput. Sci..

[27] Gil-Martín M., Montero J.M., San-Segundo R. (2019). Parkinson’s Disease Detection from Drawing Movements Using Convolutional Neural Networks. Electronics.

[28] Awatramani V., Gupta D. (2020). Parkinson’s Disease Detection Through Visual Deep Learning. International Conference on Innovative Computing and Communications.

[29] Bernardo L.S., Quezada A., Munoz R., Maia F.M., Pereira C.R., Wu W., de Albuquerque V.H.C. (2019). Handwritten pattern recognition for early Parkinson’s disease diagnosis. Pattern Recognit. Lett..

[30] Chakraborty S., Aich S., Seong-Sim J., Han E., Park J., Kim H.C. Parkinson’s Disease Detection from Spiral and Wave Drawings using Convolutional Neural Networks: A Multistage Classifier Approach. Proceedings of the 2020 22nd International Conference on Advanced Communication Technology (ICACT).

[31] Moshkova A., Samorodov A., Ivanova E., Fedotova E. High Accuracy Discrimination of Parkinson’s Disease from Healthy Controls by Hand Movements Analysis Using LeapMotion Sensor and 1D Convolutional Neural Network. Proceedings of the 2020 Ural Symposium on Biomedical Engineering, Radioelectronics and Information Technology.

[32] Shaban M. Deep Convolutional Neural Network for Parkinson’s Disease Based Handwriting Screening. Proceedings of the 2020 IEEE 17th International Symposium on Biomedical Imaging Workshops (ISBI Workshops).

[33] Moetesum M., Siddiqi I., Vincent N., Cloppet F. (2019). Assessing visual attributes of handwriting for prediction of neurological disorders—A case study on Parkinson’s disease. Pattern Recognit. Lett..

[34] Afonso L.C., Rosa G.H., Pereira C.R., Weber S.A., Hook C., Albuquerque V.H.C., Papa J.P. (2019). A recurrence plot-based approach for Parkinson’s disease identification. Future Gener. Comput. Syst..

[35] Almeida J.S., Filho P.P.R., Carneiro T., Wei W., Damaševičius R., Maskeliūnas R., de Albuquerque V.H.C. (2019). Detecting Parkinson’s disease with sustained phonation and speech signals using machine learning techniques. Pattern Recognit. Lett..

[36] de Souza R.W., Silva D.S., Passos L.A., Roder M., Santana M.C., Pinheiro P.R., de Albuquerque V.H.C. (2021). Computer-assisted Parkinson’s disease diagnosis using fuzzy optimum- path forest and Restricted Boltzmann Machines. Comput. Biol. Med..

[37] Elreedy D., Atiya A.F. (2019). A Comprehensive Analysis of Synthetic Minority Oversampling Technique (SMOTE) for handling class imbalance. Inf. Sci..

[38] Zhu T., Lin Y., Liu Y. (2017). Synthetic minority oversampling technique for multiclass imbalance problems. Pattern Recognit..

[39] Iandola F.N., Han S., Moskewicz M.W., Ashraf K., Dally W.J., Keutzer K. (2016). SqueezeNet: AlexNet-level accuracy with 50× fewer parameters and <0.5 MB model size. arXiv.

[40] Ucar F., Korkmaz D. (2020). COVIDiagnosis-Net: Deep Bayes-SqueezeNet based diagnosis of the coronavirus disease 2019 (COVID-19) from X-ray images. Med. Hypotheses.

